# Using a human resource management approach to support community health workers: experiences from five African countries

**DOI:** 10.1186/s12960-015-0034-2

**Published:** 2015-09-01

**Authors:** Joanna Raven, Patricia Akweongo, Amuda Baba, Sebastian Olikira Baine, Mohamadou Guelaye Sall, Stephen Buzuzi, Tim Martineau

**Affiliations:** Department of International Public Health, Liverpool School of Tropical Medicine, Liverpool, UK; School of Public Health, University of Ghana, Accra, Ghana; Institut Panafricain de Sante Communautaire, Bunia, Democratic Republic of Congo; School of Public Health, Makerere University, Kampala, Uganda; Department of Paediatrics, University of Dakar, Dakar, Senegal; Biomedical Research and Training Institute, Harare, Zimbabwe

**Keywords:** Close-to-community, Community health workers, Human resource management, DRC, Ghana, Senegal, Uganda, Zimbabwe

## Abstract

**Background:**

Like any other health worker, community health workers (CHWs) need to be supported to ensure that they are able to contribute effectively to health programmes. Management challenges, similar to those of managing any other health worker, relate to improving attraction, retention and performance.

**Methods:**

Exploratory case studies of CHW programmes in the Democratic Republic of Congo, Ghana, Senegal, Uganda and Zimbabwe were conducted to provide an understanding of the practices for supporting and managing CHWs from a multi-actor perspective. Document reviews (*n* = 43), in-depth interviews with programme managers, supervisors and community members involved in managing CHWs (*n* = 31) and focus group discussions with CHWs (*n* = 13) were conducted across the five countries. Data were transcribed, translated and analysed using the framework approach.

**Results:**

CHWs had many expectations of their role in healthcare, including serving the community, enhancing skills, receiving financial benefits and their role as a CHW fitting in with their other responsibilities. Many human resource management (HRM) practices are employed, but how well they are implemented, the degree to which they meet the expectations of the CHWs and their effects on human resource (HR) outcomes vary across contexts. Front-line supervisors, such as health centre nurses and senior CHWs, play a major role in the management of CHWs and are central to the implementation of HRM practices. On the other hand, community members and programme managers have little involvement with managing the CHWs.

**Conclusions:**

This study highlighted that CHW expectations are not always met through HRM practices. This paper calls for a coordinated HRM approach to support CHWs, whereby HRM practices are designed to not only address expectations but also ensure that the CHW programme meets its goals. There is a need to work with all three groups of management actors (front-line supervisors, programme managers and community members) to ensure the use of an effective HRM approach. A larger multi-country study is needed to test an HRM approach that integrates context-appropriate strategies and coordinates relevant management actors. Ensuring that CHWs are adequately supported is vital if CHWs are to fulfil the critical role that they can play in improving the health of their communities.

## Background

In addressing both the challenges of scaling up programmes to meet Millennium Development Goal (MDG) targets, universal health coverage and the post-MDG health agenda, and the shortage of formal health workers, health services and programmes have resorted to seeking much greater collaboration with communities and the use of non-formal health workers who often provide their time on a voluntary basis [1,2]. These non-formal health workers may have a single function or many. There is a wide variety of names for this kind of health worker in different contexts, for example, community distributor, community-directed health workers, health auxiliaries, health promoters, family welfare educators, health volunteers, village health workers/volunteers/teams, community health aides, barefoot doctors and community health volunteers, though they are often covered by the umbrella term of “community health worker” (CHW).

### Human resource management and CHWs

Like any other health worker, CHWs need to be supported to ensure that they are able to contribute effectively to health programmes [2-5]. Management challenges, similar to those of managing any other cadre of health worker, relate to improving attraction, retention and performance. The principles behind good management practices used with the formal health workforce could be used to inform the management and support of CHWS.

**Figure 1:**
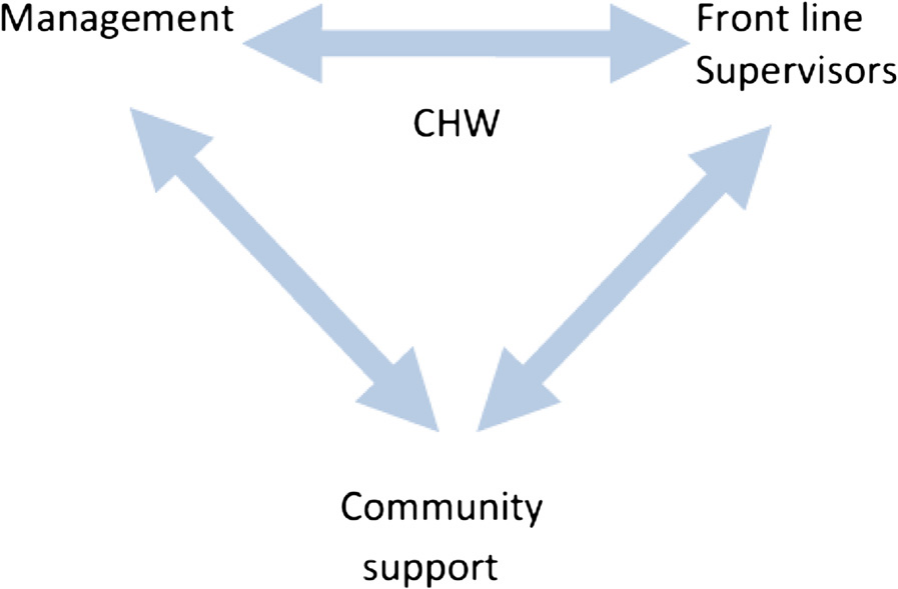
Relationships between “management” actors and CHWs.

In this study, we used the human resource management approach, as defined by Armstrong [6]: “a strategic approach to acquiring, developing, managing, motivating and gaining the commitment of … the people who work in [the organisation] and for it” [page 33]. Implicit in this concept of performance management is the link between effort and performance and mechanisms of reward and sanction [7]. To convert effort into performance requires support with direction, competencies (or development) and resources [8]. This provided the framework to guide the review of attraction, retention and performance management practices and the relationships between the management actors – front-line supervisors, programme managers and community members in CHW programmes (Figure 1).

Some CHWs are formal employees of a health organization in which salary and benefits together with incentives and disciplinary procedures are used to attract people to and retain them in jobs and to manage staff performance. However, for organizations that engage people as volunteers, the management of these challenges is somewhat different. The management practices for attracting, retaining and supporting the performance of people working on a voluntary basis or without formal contract need to be more nuanced as intrinsic motivation is likely to have greater importance. As the selection criteria often require candidates to be local, the challenges of retention may not be so great; though, if CHWs are dissatisfied, they may simply withdraw their labour without resigning. Since the volunteers are unsalaried, managers cannot dismiss them in the formal sense of the term for non-performance. Managers and line supervisors therefore have to rely on rewards rather than sanctions – which may seem unfair to formal employed workers. CHW expectations are linked to human resource (HR) outcomes such as numbers and characteristics of CHWs, length of stay as CHW and performance against job description; if expectations are met through human resource management practices, then this will affect how health workers behave in terms of being attracted to the role as CHW, staying in this role and carrying out their activities. The importance of matching performance management practices to expectations in order to influence performance was illustrated in a study of health worker motivation in Mali [9] and applies equally to CHWs. There is therefore a real need to understand CHW expectations of their role – particularly volunteers – in different contexts and the challenges and opportunities they face in their work.

### Relationship between management actors and CHWs

Community-directed programmes, in which communities are properly engaged rather than merely consulted, provide important opportunities for supporting the work of CHWs as well as some additional challenges [10,11]. CHWs can provide an important interface between programme managers and the communities they serve [12-14]. They can help to ensure that services reach the normally underserved parts of their communities, making the distribution of programme benefits more equitable. While communities can carry out some management functions (such as supplying resources, e.g. transport to enhance performance or monitoring attendance), adding another set of actors adds to the complexity of supporting CHWs – especially when the actors work in different organizational environments. From reading literature and our experience from different contexts, we have developed a figure to illustrate the actors involved in managing CHWs. The actors involved are shown in Figure 1. Health service/programme managers (“management”) will be responsible for overall programme delivery and regarding HR functions for developing practices to attract, retain and support performance of CHWs. Front-line supervisors will have more responsibility for implementing the practices – in particular, those related to performance management. Community organizations, such as a village health committee, may be involved in recruitment and selection of CHWs and some basic performance monitoring as well as helping with mobilization of people and resources to support the CHW’s work. Management and front-line supervisors may work in a bureaucratic organizational environment with performance partially driven by donor funding. Community organizations may operate to very different time frames and organizational loyalties. To maximize the support to the CHW, the three groups need to work together closely in spite of the different contexts in which they operate.

In order to be able to use CHWs more effectively in their programmes, health managers need more information on what kind of management practices (including the use of incentives) tend to work best both to attract and retain volunteer CHWs and to manage their performance. There is a need to build a picture of the range of human resource management practices currently used, how they are implemented and their reported effects on human resources, from the perspectives of the three sets of management actors and the CHWs themselves. Our plan was to carry out a rapid exploratory study to feed into the design of a large-scale in-depth multi-country study that thoroughly tests a variety of human resource management (HRM) strategies that seem to work best to attract, retain and support the performance of CHWs. This study was commissioned by WHO TDR (Special Programme for Research and Training in Tropical Diseases) who wanted the inclusion of a range of countries including Anglophone and Francophone countries, and we were therefore limited to one case study per country.

The aim of this study is therefore to use rapid country case studies to explore the current use of practices for attraction, retention and performance management of CHWs in five African countries – the Democratic Republic of Congo, Ghana, Senegal, Uganda and Zimbabwe.

## Methods

**Figure 2:**
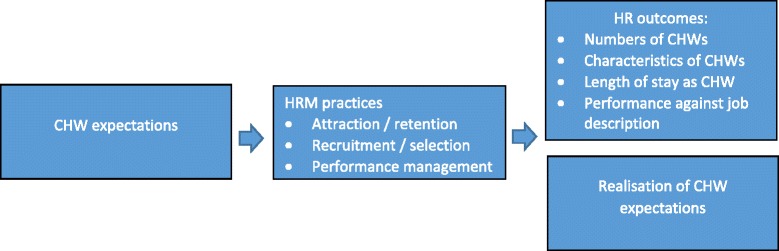
Conceptual framework of the study.

The conceptual framework of the study (shown in Figure 2) illustrates the linkages between CHW expectations, HRM practices, HR outcomes and the realization of CHW expectations. The study examined the characteristics, roles and expectations of the CHWs. It was assumed that the extent to which their expectations were met through appropriate HRM practices would determine the ability of the programme to attract and retain the CHWs and help understanding related to shortages or high turnover. The HRM practices examined included the following: attraction and retention; recruitment and selection – mainly in relation to skills potential to enable contribution to performance; and performance management of CHWs once engaged. HR outcomes were explored in terms of reported numbers and characteristics of CHWs, length of stay as CHW and performance against job description. Finally, an assessment was made of whether CHW expectations – in particular, in relation to attraction and retention – appeared to have been met. Data analysis aimed to understand HRM practices to identify areas needing improvement.

### Study design

Case studies were carried out in one district and with one programme that uses CHWs in each of the five countries. Three methods of data collection were conducted: review of documents, key informant interviews with the managers of CHWs and focus group discussions with CHWs.

### Study setting

In each country, programmes were selected using the following criteria: CHWs are a recognized cadre in the programme, CHWs are not contracted as full-time employees, CHWs are not employed on the terms and conditions of a standard paid civil service contract, there is a minimum of 30 CHWs and management involves some community direction or engagement. One district was selected in each country based on two criteria: ease of access to the study site and the country research teams have an existing relationship with the district health management team in order to facilitate access to the district, managers and CHWs. Table 1 describes the programmes and districts selected in each country.

**Table 1:** **Study programmes and districts**

**Country**	**Programme**	**District**
DRC	Expanded Programme of Immunization	Bunia district; Ituri region, North East
Ghana	Expanded Programme of Immunization and Home Management of Malaria	Atiwa district, Eastern region
Senegal	Programme Sante USAID/Sante Communautaire (PSSCII)	Rufisque, Dakar region
Uganda	Integrated Community Case Management	Masindi district, Western region
Zimbabwe	Village Health Worker programme	Mutoko district in Mashonaland, East province

### Data collection

Data collection was carried out by the country research teams and took place between October 2013 and March 2014.

#### Document review

Each country research team reviewed documents describing policies, practices, programmes and research studies related to human resource management practices used with CHWs at the national and local level. They searched ministry of health websites and offices and institutional libraries, used search engines such as PubMed and Google Scholar and consulted key informants to help identify relevant documents. A total of 43 documents were reviewed.

#### Key informant interviews

Key informant interviews (KIIs) were conducted with managers of programmes using CHWs, supervisors of CHWs and community members who are involved in the recruitment and management of CHWs. Between five and seven interviews were conducted in each country (Table 2). The interviews explored areas such as the CHW programme, responsibilities of the CHWs, human resource management practices (recruitment, attraction and retention practices, performance management, the use of incentives) and general management activities. The interviews were conducted in locations chosen by the participants such as in health centres, offices or homes and lasted between 45 min and 90 min. They were digitally recorded following permission from each participant.

**Table 2:** **Data collection summary**

**Country**	**KIIs**	**FGDs**
DRC	7 (3 supervisors; 4 community)	3 (1 male; 1 female; 1 mixed)
Ghana	7 (3 managers; 2 supervisors; 2 community)	2 (1 male; 1 female)
Senegal	5 (1 manager; 2 supervisors; 2 community )	2 (1 male; 1 female)
Uganda	5 (2 managers; 2 supervisors; 1 community)	2 (1 male; 1 female)
Zimbabwe	7 (3 managers; 3 supervisors; 1 community)	4 (1 male; 3 female)

#### Focus group discussions

Focus group discussions (FGDs) were held with CHWs who were currently working in the selected programmes. In each country, between two and four FGDs were conducted and were generally segregated by gender (Table 2). The discussions explored the experiences of being a CHW including the challenges that they faced, how they are managed and supervised, their views and experiences of incentives and any recommendations for improving the CHW programmes. They took place in health facilities and lasted between 60 min and 120 min. They were digitally recorded following consent from each participant.

### Data analysis

The country research teams completed the document review, transcribed the recordings or notes from the interviews and discussions and where necessary translated them into English. The data were analysed using the framework approach which facilitates rigorous and transparent analysis [15]. The transcripts were read and re-read to identify emerging themes; a coding framework was developed based on these themes and the conceptual framework (Figure 2), and all transcripts were coded with this framework; charts were created for all themes; these charts were used to describe similar and divergent perceptions, develop explanations and find associations between them. The computer programme “NVivo version 10” was used to support the analysis.

### Ethics

Ethical approval for the study was gained from the Research Ethics Committees at the Liverpool School of Tropical Medicine in the UK; WHO TDR; DRC Ministere de Recherche Scientifique et Technologique; Ghana Health Service Ethical Review Committee; Comite National d’Ethique pour la Recherche en Sante, Senegal; Higher Degrees, Research and Ethics Committee School of Public Health, College of Health Sciences, Makerere University, Uganda; and Institutional Review Board of the Biomedical Research and Training Institute, Harare, and the Medical Research Council of Zimbabwe. Informed consent was obtained from each participant prior to starting the interviews and discussions.

## Results

This section provides a synthesis of the findings from the five country case studies. It covers five broad areas: a description of the characteristics of CHWs as this helps us understand why HRM practices may or may not work, the broad range of activities that CHWs undertake, HRM practices of attraction and retention and how effective they are at meeting CHW expectations, recruitment and selection to get appropriate people to perform the role of CHW, performance management practices and their effect on CHW performance and finally who manages CHWs.

### Who is a CHW?

In the Democratic Republic of Congo (DRC), Senegal, Uganda and Zimbabwe, CHWs were reported to more likely be female and aged over 30 years. Several reasons were given: they are more interested in health issues, are already involved in health within families, are respected and listened to within communities and are seen as being able to work easily with people. Older women are more likely to become CHWs as they have experience of looking after children and younger unmarried women are seeking salaried employment. However, in Ghana, informants explained that CHWs are more likely to be male. As CHW work requires travelling around communities and is time-consuming, men are more likely to take up this work, while women are more occupied with taking care of their farms, homes and families.

CHWs had many expectations on working as a CHW including serving the community, enhancement of skills and knowledge, receiving financial benefits in return for the work they do, being recognized as a health worker in the community and having social status and prestige within the community and their role as a CHW fitting in with their other work such as looking after the family and home and other paid work

### What does a CHW do?

The respondents reported that each CHW undertakes a wide range of activities. These can be divided into three main categories: provision of healthcare services, health promotion or distribution of health goods and community organization. Table 3 describes the range of activities of CHWs across the five country contexts. CHWs reported the activities they undertake, and while each CHW reported activities from all of the categories, each CHW did not necessarily carry out all of the activities. CHWS from all study contexts reported that their workload is very heavy. For example, in Senegal, the CHWs reported that they work at the health centres in the morning and do home visits during the afternoon. In Zimbabwe, they reported that when they started working as a CHW (on average 10 years ago), they worked only on the malaria programme, but now, they are expected to work on all programmes in the district. In Uganda, CHWs explained that they found it difficult to undertake all activities as their catchment area is large, and therefore, they have greater distances to travel.

**Table 3:** **CHW activities**

**Provision of healthcare services**	**Health promotion/distribution of health goods**	**Community organization**
• Home-based treatment of illnesses such as malaria, diarrhoea, ARI	• Home visits to promote health	• Encouraging community to undertake specific tasks such as setting up ambulance service, addressing specific health issues, collecting water for the health centre and health facility maintenance/repairs
	• Health education on areas such as HIV/AIDS prevention, nutrition, breastfeeding and hygiene	
• Follow up of TB/HIV patients		
• Assisting in clinics (weighing, record keeping)	• Providing nutrition/cookery classes	
• Providing health education in health facilities	• Expanded Programme of Immunisation: defaulter tracing, mobilization of communities	
		• Advocating for community ownership of programmes (for example, support to CHO and CHPS programme in Ghana)
• Collecting data: community register and disease surveillance		
	• Promoting health insurance scheme	
• Attending planned and emergency births		
		• Representing community at meetings (health committee, development committee)
	• Distributing and checking use of bed nets	
• Providing antenatal and postnatal care services		
	• Mass drug administration – distribution, house-to-house visits (annual)	• Linking with other volunteers (for example, working with other NGOs)
	• Family planning education and contraceptive distribution	
	• Stimulating demand for and raising awareness of formal health services/referrals	
	• Referrals for complications in pregnancy, illnesses in under 5s, HIV VCT, burns/injuries	

CHWs do not have fixed schedules of working, apart from when there are immunization or mass drug administration (MDA) campaigns. They can be flexible in when they work and try to fit their CHW activities with their farm and home work. However, most reported that community members visit them at any time of the day or night, and therefore, they tend to work on most days. Some CHWs reported that the heavy workload interfered with their income-generating activities and farm work.

### HRM practices: attraction and retention

HRM practices related to attraction and retention employed in the five country contexts affect if and how expectations (as described earlier) are realized and which further influence the behaviour of the CHWs in relation to being attracted to the role of CHW and staying as a CHW. Table 4 provides a synthesis of the expectations, HRM practices and HR outcomes across all the country contexts but does not attempt to demonstrate causality from this exploratory study. It was not possible to attribute individual HRM practices to HR outcomes. Across all contexts, skills and knowledge enhancement was a key expectation of the CHWs, which was partially realized through the initial and refresher training that they received. However, CHWs reported that refresher training should be more frequent and of better quality to better meet the needs of their job. CHWs also expected to receive some financial benefits in reward for the work they did, such as stipends, training per diems and travel allowances to meetings and trainings. However, this expectation was rarely met, as the stipends were seen as inadequate in relation to the amount of work done and there were delays in receiving them. There were also problems with the training and travel allowances: CHWs rarely received them, and when they did, they did not cover the cost of transport. Participants from all contexts reported that there were no guidelines on incentives for CHWs. Another key expectation was that the CHW role should fit in with income-generating activities and family commitments. This was also not generally met. In Ghana, for example, CHWs had job descriptions outlining their role and responsibilities and had some supervision from health centre staff, but they rarely received help with farming work and so struggled to support their families. CHWs also reported that their role should enhance their social status. However, CHWs were not always valued by their communities (for example, in Ghana, where CHWs reported feeling undervalued by their communities as help with farming that was promised was not given) and not appreciated by the health facility staff. There were also issues with the provision of items that aided their recognition as health workers and improved their status such as uniforms, badges and t-shirts. They were not given (DRC), they were provided only when CHWs were recruited and not replaced (Uganda, Zimbabwe, Ghana) and the uniform sizes were too small and the CHWs were too embarrassed to wear them (Zimbabwe). Two more detailed examples are given below: one where CHW expectations have been met by HRM practices and one where expectations have not been met.

**Table 4:** **Attraction and retention: CHW expectations, HRM practices and outcomes**

**CHW expectations**	**HRM practices**	**Expectations met**	**HR outcomes**
*Skills and knowledge enhancement*	• Initial and refresher training	• Further improvement wanted: frequency and quality of refresher training	• Most CHWs are female
			• CHWs more likely to be older
• Enhancing skills for main role	• Opportunities for further training		
			• Shortage of CHWs
			• Many candidates apply to be CHW in Zimbabwe
• Enhancing health skills to serve families at home			
			• Average length of service: 8–10 years
*Financial benefits*	• Provision of financial incentives	• Irregular/insufficient per diems and transport reimbursements	• Few CHWs leave
• Per diems for training and other events			• Younger CHWs are more likely to leave; leave for paid jobs; young women leave when they marry
	• Free/reduced fees for healthcare for CHWs and families		
• Fixed stipends – per time worked or per activity		• Stipends: inadequate amount; delays in receiving stipends	
	• Incentives from health campaigns, for example, immunization		
		• No written guidelines on incentives for CHWs	
	• Lunch and travel allowance for meeting attendance		
*CHW role fits with other roles*	• Exemption from communal labour; help with farming	• Use of job description varied: job described in training or CHW has job description or no job description	
• Manageable with other job			
	• Use of job description		
• Manageable with other responsibilities such as farm work, looking after home and family		• Irregular supervision by health centre supervisors	
	• Supervision: reporting to supervisors; regular meetings with supervisors; supervisory visits to community		
		• Supervision does not monitor workload	
		• Community support for farm work often lacking	
*Social status and prestige*	• Provision of t-shirts, uniforms, badges, etc. to aid recognition as health worker	• CHWs not always valued in community	
• Seen as a “Doctor” – community status and respect		• CHWs not always supported/respected by health staff	
• Recognition as a health worker	• Recognition by the community; official ceremony when CHWs are recruited	• Lack of incentives such as t-shirts, ID badges, equipment for gardening (formerly provided by NGOs)	

The DRC case study provides a good example of how HRM practices helped to ensure CHW expectations were met. They expressed the desire to serve their community through providing health support especially for children and helping with access to healthcare services. They received feedback from their supervisors, so they knew they were doing the right things and received “management” support and recognition from the community. As a result, most feel that their desire to serve the community effectively is fulfilled.

“It is the community which supports us. When we arrived in the community, there are children already prepared by the parents to be vaccinated. This is our conclusion.” (CHW, FGD, female, DRC).

“When we send a patient to the health centre for medical care and leaves the centre being cured, it is our great joy and honour in the community. We are happy because God has blessed our work.” (CHW, FGD mixed, female, DRC).

In contrast, in Zimbabwe, CHWs reported expecting to receive remuneration for the work that they do. CHWs are supposed to receive a regular stipend of $14 per month, but CHWs reported several issues with this practice. They receive the stipend irregularly. They spend a whole day at the district headquarters, without food, waiting for the payment. They also perceive the amount as too small compared to the work that they do, and particularly, as they have to pay up to $6 in transport costs in order to collect it. Finally, the stipends are dependent on donor funding, so when the funding is not available, then the CHWs do not receive the stipends.

“You wait for long to get that $42 dollars which sometimes comes after 7 months. They make us wait for each other till everyone comes. At one time we had to wait for 3 days. We use our money to get transport to and from Mutoko Centre and to buy food.” (CHW, FGD, female, Zimbabwe).

“We were told that when you become a CHW you will get an incentive so I thought it would help me and my family.” (CHW, FGD, male, Zimbabwe).

### HRM practices: recruitment and selection

Selection and recruitment of CHWs appears to be a complex process with several mechanisms implemented even within the same contexts. Participants reported that community members volunteer to be CHWs, and they then go through a selection process either by being interviewed by village leaders or councillors or by being selected by the community. Communities also nominate several potential CHWs, and either the community leader selects or the community votes for the CHWs. In some settings, the health authorities ask community members to be CHWs.

**Figure 3:**
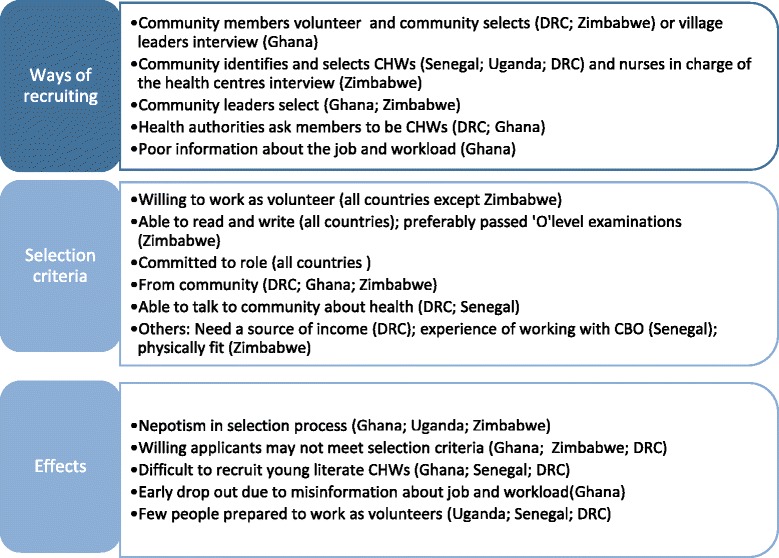
Selection processes, criteria and effects.

There were several selection criteria that were common across the contexts which included the following: ability to read and write, being committed to the role of CHW, being from the community and ability to communicate with the community about health. In DRC, they also reported that having a source of income was an important selection criterion. The respondents described several negative effects with the way that CHWs are selected: nepotism in the selection process; willing applicants may not meet selection criteria; many candidates, particularly older ones, do not meet the educational criteria and, related to this, the difficulty in recruiting young literate CHWs; early drop out due to misinformation about job; and few people volunteer. Figure 3 provides details of the selection process, criteria and the effects across the five countries.

“The challenges we sometimes face is that you just do not find someone who knows how to write and to read. But you end up selecting someone committed to help, but having difficulties to read and to write. I think that is the major challenge.” (KII, community member, male, DRC).

“There are challenges when recruiting CHWs. Few people are ready to apply for the work. Young people aged 18–22 years are very difficult to be recruited as they look for paid jobs. There is lack of financial motivation.” (KII, community member, female, Senegal).

“The other challenge with these things is political and we don’t want the chairmen to select but they were forcing their way in to select their own people. You find a man selecting a wife and a son, and then you are asking the other community members to select, they select different people and this one would annoy the chairpersons and when this annoyance has continued you find the chairpersons unhappy with the CHWs because his people were left out.” (KII, supervisor, male, Uganda).

### HRM practices: performance management

The study showed several HRM practices that influence performance in many of the country contexts. They include initial and refresher training, use of job description and supervision. There were also several practices conducted in individual settings such as provision of awards for good performance, review workshops to check competencies and dismissing volunteers. However, a number of gaps in practices were also identified. Common across many contexts were lack of necessary equipment, drugs and supplies to carry out their role; lack of transport or financial support for travel around the community and to meetings; insufficient training or supervision to keep skills up to date; and lack of support from community. In a few contexts, other gaps were reported such as irregular supervision, no job descriptions and no feedback to CHWs following evaluations. Table 5 provides an overview of the HRM performance practices conducted and the gaps in supporting performance.

**Table 5:** **HRM performance practices across the five country contexts**

**HRM practices that appear to support performance**	**Gaps in supporting performance**
*Practices reported across many contexts*	*Gaps reported across many contexts*
• Initial training: length and topics varied, but some dissatisfaction with length and coverage	• Lack of equipment, drugs and supplies
	• Lack of transport or support for travel
• Refresher training; *ad hoc*, related to programmes or linked to meetings; but some dissatisfaction including lack of allowances and frequency	• Skills not kept up to date e.g. insufficient training for multiple roles
	• Lack of support from community members / community’s unrealistic expectations of what CHWs can do
• Job description: job described in training; job description given to CHW	
• Supervision: send reports to supervisors; regular meetings with supervisors; supervisory visits to community	*Gaps reported in one context*
	• Lack of regulation of CHW practice vs training given (Senegal)
*Practices reported in one context*	• Irregular supervision by health centre supervisors (Uganda)
• Annual performance awards nominated by community health officers (Ghana)	
	• No job descriptions (Uganda)
• Cash reward for identifying a case of guinea worm (Ghana)	• External evaluators observe immunization and report to health centres but not to CHWs (DRC)
• Community can sack a volunteer, if he/she does not carry out their duties (Ghana)	
• Some review workshops to check competencies (Zimbabwe)	

Two more detailed examples of performance practices and their effects on CHW performance are now provided. The Ghana case study provides a good example of where performance practices are linked to good performance. Initial training was provided to CHWs before they started work, and this covered their roles, clinical topics and how to work with communities. Skills were further developed through in-service training provided on an *ad hoc* basis – but may also be timely in relation to skills needed for particular activities – and through regular meetings with supervisors. Respondents reported that these performance-management-related HR practices of initial and in-service training as well as regular meetings with supervisors helped to provide a wide range of services, manage a heavy workload and improve the health of communities.

“The way we receive training and respect for us from our authorities, it encourages us to work harder and give out our all for the health of the community.” (CHW, FGD, male, Ghana).

“They are in the community and are likely to save these people and give report or warning on any symptoms of TB, or guinea worm case or even treatment compliance issues. These occur more in the community than in the hospitals. When daily treatment is given by visiting the nurses daily, many default. But when volunteers are used to supervise the compliance to treatment by the community, it really works. Volunteerism needs to be sustained.” (KII, manager, Ghana).

In contrast, in Uganda, there are performance problems which can be linked to the absence of performance-related practices. CHWs and key informants reported a lack of equipment, drugs and materials to provide care. This resulted in CHWs being unable to assess patients and provide treatment, referring patients to facilities when they could have been treated at home and communities having negative perceptions of the CHWs.

“The biggest problem is the drugs. If the supplies can be constant, that is one of the motivations of CHWs by the way. When drugs are there, the community is happy. They even call them doctors.” (KII, manager, female, Uganda).

“The challenges include: shortage or complete lack of medicines, and equipment to use. Patients come at night and we do not have drugs and even a torch to help us check them.” (CHW, FGD, female, Uganda).

### Who manages the CHWs?

We examined the role of the different management actors – health service/programme managers, front-line supervisors and community organizations – in managing CHWs in the five country contexts. There is some involvement of district-level programme managers in the management of CHWs, in Ghana, Uganda and Zimbabwe. For example, in Uganda, a member of the District Health Management Team is responsible for supervising CHWs. In addition, there is a national programme manager in Zimbabwe who develops the handbook, organizes logistics and training and visits VHWs in the field. In Senegal and DRC, there appears to be very little contact between the CHWs and programme managers; however, some direction and support to CHWs from the community is provided through health centre committees. In Ghana, for example, the committee meets every month where all health issues are discussed and the activities of all health workers including CHWs are planned. However, in some contexts such as DRC, Uganda and Zimbabwe, this support is minimal. Most of the supervision and management happens at the health centre level, where the chief nurses or community health officers in the case of Ghana supervise and manage the CHWs. They play an important role in the motivation and support of CHWs. CHWs explained that they report to the health centre staff about their activities either through written reports or verbally at meetings, and the nurses provide feedback which they valued. Nurses and community health officers reported that they do supervision visits with the CHWs, but the frequency of visits varied across the five contexts due to time and transport constraints. In Uganda and DRC, senior CHWs also take on this role. In DRC, a senior CHW heads a community outreach group of CHWs and holds regular meetings to discuss activities and solve problems. This group then reports to the health centre nurse. In Uganda, the village health team coordinator (a senior CHW) visits the CHWs to provide support and advice and help with any problems. Most CHWs viewed the supervisors as being supportive and felt valued and respected. However, in DRC and Zimbabwe, some felt they were not supportive and did not provide the necessary resources to do their work.

## Discussion

The study revealed that CHWs had many expectations of their role in healthcare, including serving the community, enhancing skills and knowledge, receiving financial benefits in return for the work they do, having social status within the community and their role as CHW fitting in with their other responsibilities. Many of the normal HRM practices are employed, but how well they are implemented, the degree to which they meet the expectations of the CHWs and their effects on HR outcomes vary across contexts. Front-line supervisors, such as health centre nurses and senior CHWs, play a major role in the supervision and management of the CHWs and are central to the implementation of the HRM practices. On the other hand, in our case studies, community members and programme managers have little involvement with managing the CHWs.

There are several limitations to this study. This exploratory study was designed to be carried out rapidly to provide a snapshot of what support was being provided in a range of CHW programmes in one location in five different countries. The speed and breadth was at the expense of the detail including on the management structures and processes and context. However, we do not claim that the findings can be generalized to all contexts. Also, this did not allow for detailed investigation of HRM practices including constraints to using them and attribution of the impact on HR outcomes. Because of the limited size of the study, we did not include views of service users, and the short duration of the study precluded following up people who had left the CHW role. While questions were asked about incentives, the subject of a forthcoming systematic review [16], neither managers nor CHW provided clarity on the purpose of these or how they were administered. The analysis therefore focused more on broad HRM practices used – some of which included incentives. Another limitation of this study is that we did not explore the gender dimensions of CHW expectations and management.

### The use of an HRM approach in supporting CHWs

Since the recognition of an almost global shortage of health workers in the previous decade, there has been a resurgence of interest in the use of community health workers to deliver health services. CHWs, like any other health worker, need support to deliver services effectively [17]. Our study looked at the support provided to CHWs in single programmes in five African countries through a HRM lens and multi-actor perspectives. This was because the areas included in HRM described by Armstrong above [6] (acquiring or attraction and recruitment; developing through pre-service and in-service training; managing, motivating and gaining the commitment to improve performance and to retain staff) are all highly relevant to the roles and contributions of the CHWs in the five contexts covered by this study – and presumably in every other context.

The challenge in HRM is to integrate the needs of the organization – in this case, the CHW programme – with the personal needs or expectations of the worker [18] – in this case, the CHW. In the context of formal employment, a major factor in meeting the personal needs of the work is pay which acts as a form of extrinsic motivation [19] – something that by definition a volunteer does not receive. Nevertheless, the study shows that there are indirect financial benefits [20] that could or do make volunteering attractive such as money that can be saved from training per diems, a fee for specific tasks (for example, for immunization campaigns), help with paying health insurance contributions and free or subsidized healthcare treatment. In the context of low levels of employment in the formal sector, gaining experience working as a health volunteer and learning skills on the job is seen as strategic for entering into professional health worker training in order to get more secure and better paid employment.

For volunteer CHWs, other less tangible benefits – such as job satisfaction in helping fellow citizens, fulfilling socio-cultural obligations or being respected as a health worker – play a much more important part in meeting the personal needs of the worker. The “psychological contract” suggests that intrinsic motivation (coming from the job itself) is important for salaried employees but takes a far more important role in the absence of pay [21].

The HRM approach involves examining all the expectations of health workers – salaried or volunteer – to identify to what extent they can be met through an integrated set of HRM practices relating to attraction, recruitment, retention and performance management (including the provision and maintenance of adequate competencies) while at the same time achieving the goals of the organization or programme. It can therefore be concluded that the HRM approach normally used for salaried staff is also appropriate for volunteer CHWs but requires adaptation to the specific contexts and characteristics of the CHWs.

A final and crucial aspect of HRM strategy is that the various practices for attraction, retention and performance management are aligned horizontally to ensure that they complement each other – training is supported by follow-up supervision, for example [22]. Lack of alignment of HRM practices may lead to conflict or the practices undermining each other and workforce problems may not be effectively addressed or there may even be negative unintended consequences. Horizontal alignment of HRM practices will only be achieved through a coordinated approach to achieving an HRM strategy [23].

In this study, we found HRM practices related to all areas of attraction, recruitment, retention and performance management. In some cases, HRM practices were achieving the desired HR outcome. For example, with the exception of Uganda, CHWs interviewed had been “in post” for 8 to 10 years on average (which would be considered very effective staff retention in the formal sector). This may be because in the four countries, CHWs were reported to more likely be female and aged over 30 years and assuming they were local and therefore comprising a group more easily retained especially in conservative rural societies. In some cases, the practice was intended but not implemented, for example, help with farming to free up the time to carry out healthcare work in Ghana. Sometimes, the practice was in place but considered inadequate. For example, incentives for carrying out health campaigns in DRC were too small and too late and therefore not sufficient to achieve the desired performance. There were also cases where the expectations of the CHWs, mostly local, at the time of recruitment had hardly been met. HRM practice by managers had not sufficiently controlled the workload to enable CHWS to carry out their personal tasks such as farming, causing some CHWs wanting to leave. There is a lack of good evidence on how much time CHWs spend on their CHW work and how this affects their livelihoods, as well as how CHWs allocate this time to different health activities [24]. Understanding CHW workload and use of time is important for effective management. However, there are many challenges in carrying out this type of study.

Programmes appeared to benefit from the widespread commitment to providing healthcare for the CHWs’ fellow citizens. This intrinsic motivation may have been masking some of the inadequacies of HRM practice. In this brief study, we were unable to attribute clear links between HRM practice – and differentiate between practices addressing intrinsic and extrinsic motivation – and HR outcomes.

Clearly there is room for improvement in the HRM practices being used in the case study programmes. But investment would not be fully justified without a coordinated approach to ensure horizontal alignment of improved HRM practice across all key areas.

However, there was no evidence of such coordination of HRM practices for the CHW programmes in the case studies, though as mentioned above this was not investigated in great depth. Integrated HRM is frequently absent in the formal health sector and especially in lower and middle income countries [25]. The formal health sector often has the problem of dual management – where there is an administrative line of supervision through the main health service structure and an additional supervision structure for specific programmes – which means the health worker sometimes has to choose which instructions and advice to follow. With CHW programmes, the situation is even more complex if the aim is to gain ownership by the communities being served through the provision of a significant role for community representatives in programme and staff (CHWs) management [2]. The often-quoted risk of engaging community representatives in management of CHWs was borne out in the claims of nepotism in three countries. However, this lack of alignment of objectives may just as easily happen at higher levels of the system [26].

### The need for an HRM approach

Given the exploratory nature of the research, it would be inappropriate to make recommendations about specific HRM practices to support CHWs. Furthermore, the literature is unclear on the benefits of some of these practices, for example, the use of incentives [27], or provides specific guidance, for example, on supervision and appraisal [28]. In addition, the practices need to respond to the specific context of the programme [29,30]. Several authors have promoted a set of HRM practices (for example, using coordinated mix of HRM practices including supervision, incentives, recruitment and training [31]) or, with more focus on productivity, paying attention to workload, supportive supervision, supplies and equipment and respect from the community and the health system [32]. We have found that a strategic HRM approach is applicable and necessary to the support of CHWs combining an integrated set of HRM practices. First, a clear definition of the roles of the CHW is needed, along the lines of Table 3. The various management actors then need to be identified and human resource management roles clearly established. As shown in this study, there may be more than the three types of managers identified in Figure 1, and areas where the study found gaps in management (for example, that from community support) could be avoided. Coordination of the different management roles would be a challenge, but this is essential to ensure a coherent and strategic HRM approach to supporting their CHWs. Many managers are already used to managing staff on different types of employment arrangements (for example, permanent employees and those on contract) with different emphasis on HR practices. It will be a further challenge to co-manage volunteers with community representatives, but their generic skills can be applied. The management actors first need to understand the general expectations of the CHWs when they join, or in cases of shortage of recruits, the expectations of those who would join if the conditions were better. With that knowledge, managers could design a specific set of HRM practices that meet the expectations of the new recruits as far as possible while ensuring the CHW programme meets its goals. It will also be important to take gender into account when designing and implementing HRM practices. In a recent review of the evidence of how context influences the performance of CHWs, gender was an important factor [29]. Female CHWs may struggle with legitimacy, need to negotiate approval to do voluntary work from family members and may encounter problems with mobility and communication because of societal gender norms and roles [33]. Managing expectations, that is, being realistic about which expectations are likely to be met, is an important part of HRM practice. A “job preview” will be needed to manage expectations at the recruitment.

It will then be important for the management actors to monitor, as with any other area of management, the implementations of the HRM practices in terms of appropriate implementation and HR outcomes. It will not be possible to be sure which HRM practices will work, and over time, expectations may change. In either case, the HRM practices may need to be changed. With researchers working alongside managers, more useful knowledge will be generated about the attribution of certain HRM practices – or bundles of practices [34] – that lead to better HR outcomes with CHWs, thus providing them with much needed support.

Further testing of this approach is needed. Researchers can work with programmes that are looking to improve CHW performance. HRM of CHWs, specific to the unique requirements of volunteers, can then be integrated into the existing management system. Evaluation of such an approach would focus on the changes in HR outcomes for CHWs, but process monitoring, including the constraints to implementation of each HR practice and the roles of different types of managers, would be essential to explain these changes.

## Conclusions

This study explored the current use of practices for attraction, retention and performance management of CHWs in single programmes in five African countries. In these settings, we found that CHW expectations are not always met through the HRM practices. While there is substantial literature on the application of individual HRM practices for CHWs and several papers recommend integrating these strategies, this paper calls for a coordinated HRM approach to support CHWs, whereby HRM practices are designed to not only address expectations but also ensure that the CHW programme meets its goals. There is a need to work with all groups of management actors (programme managers, supervisors, community-level managers and others involved) to ensure the use of an effective HRM approach. A larger scale multi-country study is needed to test an HRM approach that integrates context-appropriate strategies and coordinates relevant management actors. Ensuring that CHWs are adequately supported is vital if CHWs are to fulfil the critical role that they can play in improving the health of their communities.

## Abbreviations

CHW: Community health worker
DRC: Democratic Republic of Congo
FGD: Focus group discussion
HR: Human resources
HRM: Human resource management
KII: Key informant interview
MDG: Millennium Development Goal
NGO: Non-governmental organization
